# Novel endoscopic resection of synchronous dual gastric lesions using a compact robotic arm system

**DOI:** 10.1055/a-2765-5732

**Published:** 2026-01-22

**Authors:** Haoran Liu, Hanchao Pan, Wendao You, Dongtao Shi, Siyue Zhang, Rui Li

**Affiliations:** 1 74566 Department of Gastroenterology, First Affiliated Hospital of Soochow University, Suzhou, P. R. China

Conventional endoscopic submucosal dissection (ESD) is challenged by poor visualization due to insufficient traction, raising risks of perforation or bleeding. Robotic systems have emerged to address these limitations, offering improved stability and precision 1 . However, most existing platforms require dedicated double-channel endoscopes or occupy the working channel, increasing complexity and deviating from standard endoscopic workflows. We report the first successful case of robot-assisted ESD using EndoFaster, a compact robotic arm system that mounts externally to standard endoscopes without modifying the scope or occupying the instrument channel.

**Fig. 1:**
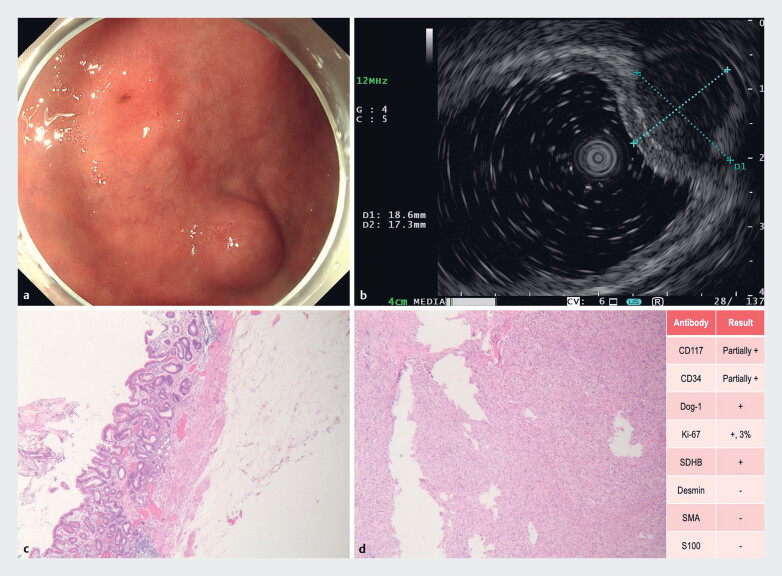
Endoscopic appearance and histopathological images. **a** Gastroscopy revealed a 0-IIc-type lesion (approximately 13×10 mm) at the anterior wall of the gastric body and an adjacent submucosal tumor. **b** Endoscopic ultrasonography demonstrated a hypoechoic lesion (18.6×17.3 mm) at the anterior wall of the gastric body, originating from the muscularis propria layer with heterogeneous internal echogenicity. **c** Pathology of the gastric body lesion revealed moderate chronic active gastritis with intestinal metaplasia, focal high-grade intraepithelial neoplasia, and lymphoid follicle formation (HE, 4×). **d** Pathological examination of the submucosal tumor revealed a spindle cell neoplasm (HE, 10×), consistent with gastrointestinal stromal tumor (GIST) based on immunohistochemical findings.

A 73-year-old male presented for endoscopic intervention following gastroscopic identification of synchronous lesions ( Fig. 1 **a** ): a 0-IIc-type lesion at the anterior wall of the gastric body (histologically confirmed as high-grade intraepithelial neoplasia) and an adjacent submucosal tumor (EUS-diagnosed as intraluminal-extraluminal protruding gastrointestinal stromal tumor, Fig. 1 **b** ). Due to the anatomical proximity of these lesions where conventional surgery would mandate a partial gastrectomy, our team elected to perform robot-assisted ESD, prioritizing both oncological efficacy and organ preservation.

**Fig. 2:**
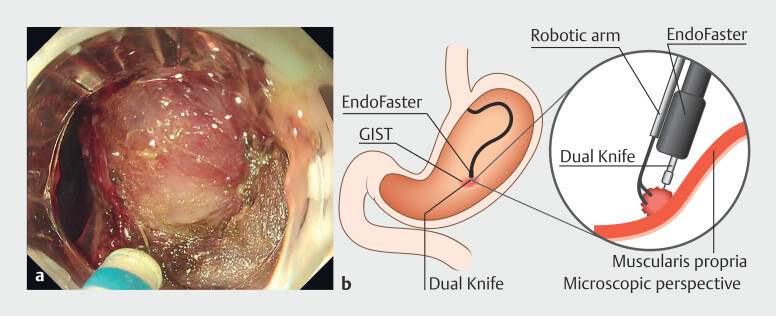
EndoFaster’s traction converted the trans-mural GIST to an intraluminal profile, enabling precise intermuscular dissection with the DualKnife. **a** Endoscopic view. **b** Schematic illustration.

Following circumferential incision of the gastric body lesion, the EndoFaster (ROBO Med, Shenzhen, China) robotic wrists provided dynamic traction while the DualKnife (Olympus, Tokyo, Japan) facilitated precise dissection along the submucosal plane. The robotic system's enhanced maneuverability enabled exclusive forward-view dissection without additional submucosal injections, achieving en bloc resection within 30 minutes while leaving the muscularis propria unharmed. For the submucosal tumor, robotic traction optimally exposed the tumor-muscularis interface, allowing selective dissection in the intermuscular space and avoiding full-thickness resection ( Fig. 2 ). The entire procedure was completed in 46 minutes without perforations ( Video 1 ). The patient recovered uneventfully and pathology confirmed R0 resection for both lesions ( Fig. 1 **c** , Fig. 1 **d** ).

Novel robot-assisted endoscopic resection of synchronous dual gastric lesions using EndoFaster.Video 1

This case demonstrates the clinical value of endoscopic robotic systems in managing anatomically challenging or multifocal gastric lesions, potentially expanding indications for endoscopic therapy and shifting paradigms in gastrointestinal oncology.
